# Case studies on community care in Japan: considerations for mitigating social isolation and loneliness in older adults with dementia

**DOI:** 10.3389/fpubh.2024.1411217

**Published:** 2024-11-22

**Authors:** Li-Mei Chen, Megumi Inoue, Nina Buckley

**Affiliations:** Department of Social Work, College of Public Health, George Mason University, Fairfax, VA, United States

**Keywords:** social capital, community-level intervention, dementia, community social work, dementia café, social isolation, loneliness, case study

## Abstract

This article explores dementia care in Japan’s aging population with a focus on mitigating social isolation and loneliness in older adults with dementia. Through an in-depth examination of case studies, the study highlights several community-based interventions, including Community Cafés, the Dementia Supporter Caravan, and the Omuta City Dementia Care model. These cases provide insights into how these initiatives foster community engagement and inclusive environments. Using a socio-ecological (SE) framework, the analysis focuses on the effectiveness of leveraging social capital to address the social challenges faced by people living with dementia (PLwD) and their caregivers. The case studies emphasize context-sensitive strategies tailored to Japan’s cultural and demographic landscape, offering lessons for reducing isolation and promoting community support for older adults with dementia.

## 1 Introduction

As populations around the world age, public health systems face increasing challenges, with dementia becoming one of the most pressing issues. The World Health Organization (WHO) estimates that 55 million people worldwide live with dementia, a number projected to reach 139 million by 2050 due to rising life expectancy (1). Dementia affects cognitive and functional abilities and contributes significantly to social isolation and loneliness, particularly among older adults. These issues can negatively impact mental and physical health, heightening the risk of morbidity and mortality. In response, initiatives like WHO’s Global Action Plan on Dementia (2017–2025) prioritize addressing social isolation among people living with dementia (PLwD) (1).

Japan is uniquely positioned at the forefront of these demographic shifts, with 29.1% of its population aged 65 or older and this figure projected to rise to 34.8% by 2040 (2). Dementia prevalence is also increasing, with one in five people aged 65 or older expected to have dementia by 2025 (3). These trends present considerable public health challenges, particularly concerning the social isolation and loneliness that often accompany dementia. Addressing these issues requires a multi-faceted, community-based approach that takes into account the specific cultural, social, and environmental contexts in Japan (4).

Social isolation and loneliness are distinct but related phenomena. Social isolation is an objective state of limited social contact (5, 6), while loneliness is a subjective experience of emotional distress due to a perceived lack of connection (7, 8). In Japan, urbanization, family structure changes, and cultural factors exacerbate social isolation. Traditionally, older adults were supported in multi-generational households, but now, increasing numbers live alone, especially in urban areas, where fast-paced lifestyles weaken social ties (9). In rural areas, isolation is exacerbated by fewer services and depopulation, leaving many older adults without adequate support networks.

Culturally, Japanese society places strong emphasis on personal dignity, self-reliance, and stoicism, discouraging individuals from seeking help for loneliness (10, 11). Stigma surrounding dementia and mental health issues further inhibits social participation and support-seeking behavior. For instance, dementia was historically associated with derogatory terms, contributing to the marginalization of individuals with the condition (12, 13). Dementia is currently written as “cognitive disorder” in kanji (Chinese characters in Japanese writing), but a different term was previously used in Japan that included a derogatory kanji referring to foolishness or stupidity (14). Such stigma and social rigidity can significantly impact the allocation of social welfare and public health resources, creating challenges for people with dementia and their families. The Japanese term “ikizurasa,” reflecting the psychological burden of living with such challenges, underlines the multidimensional nature of loneliness and social isolation, further compounded by stigma, discrimination, and other socio-ecological adversities (15). These stigmas and cultural expectations shape how social isolation and loneliness are experienced in Japan, adding complexity to efforts to address them.

Given the severe impact of social isolation and loneliness on health, it is essential to design interventions that address these issues at a systemic level. This paper explores the socio-ecological (SE) approach, focusing on its potential to mitigate loneliness and isolation among PLwD in Japan by leveraging community resources and social capital.

### 1.1 The socio-ecological (SE) approach: a comprehensive framework for addressing loneliness and social isolation among PLwD

To address the profound social consequences of dementia, Japan has adopted a two-pronged strategy: legal protections for people with dementia, exemplified by the Basic Act on Dementia (16), and a socio-ecological (SE) public health approach at the community level. The SE approach emphasizes the importance of building social capital to combat the unique challenges faced by PLwD and their caregivers (17). Social capital refers to the networks, norms of reciprocity, and trust that facilitate cooperation within communities, and it plays a crucial role in enhancing well-being and reducing social isolation.

The SE framework is interdisciplinary, integrating insights from various fields to analyze how individual, community, and policy-level factors interact to influence health outcomes (18). For example, in public health and community planning, the SE approach identifies factors at individual, community, and policy levels that influence behaviors such as physical activity and social participation. Interventions within this model consider these multilevel influences to promote health and well-being effectively. In mental health services, the SE model emphasizes the importance of multi-stakeholder partnerships and community-level participation to address access barriers and social determinants of health. Additionally, the approach advocates for preventive measures and community-based initiatives that address broader causal factors such as creating environments that enhance quality of life through integration with nature (19) or designing public spaces like parks that encourage physical health and community engagement (20). In the context of dementia care, SE emphasizes the role of community-based initiatives, preventive measures, and partnerships in improving quality of life for individuals and communities alike. This approach highlights the importance of local governance and collective action, particularly in Japan, where community-driven models such as chiiki-ryoku (CR) have proven effective in addressing social isolation and building resilience.

### 1.2 Introducing Chiiki-ryoku: a culturally grounded model

While the SE approach provides a comprehensive framework for understanding the multi-level factors affecting health, Japan has developed a culturally specific enhancement known as Chiiki-ryoku (CR). CR comes from a combination of the term “chiiki,” which translates to ‘region’ or ‘community’, and “ryoku,” which translates to ‘power’ or ‘capacity’. Rooted in Japanese community and regional development, CR emphasizes local governance, community capacity building, and resilience. It incorporates a culturally nuanced understanding of social capital – defined as networks, norms of reciprocity, and trust that facilitate cooperation among community members (21) – to enhance community well-being and address social isolation.

CR is built on three essential dimensions (22): (1) fostering community interest and engagement, where all members actively participate and show genuine concern for their local environments; (2) accumulating local resources, which entails building robust local living environments and establishing community organizations that support residents; and (3) cultivating community self-governance, encouraging active resident participation in local events and fostering community-led initiatives. This dual focus on “soft” factors such as resident attitudes, activities and behaviors, and “hard” factors, like community resources and governance structures, enables communities to harness local knowledge, volunteerism, and civic engagement to foster a higher level of social consciousness and problem-solving capacity (23).

While CR is deeply embedded in Japanese culture, its principles have broader applicability. Other countries have adopted similar community-focused models, demonstrating the potential for CR to be adapted to different cultural contexts. For instance, community currency systems in Thailand and the United States share CR’s core objectives of fostering local engagement and resilience (24, 25). The integration of CR within the SE framework offers a unique model that combines community capacity-building with a holistic approach to public health.

The integration of CR within the SE approach offers a unique model that goes beyond traditional public health frameworks by emphasizing the role of community capacity and local governance in enhancing social capital. It aligns closely with the SE model’s emphasis on multi-level, transdisciplinary approaches by incorporating both individual-level initiatives (such as promoting civic engagement and social participation) and community-level strategies (such as building supportive environments and social networks). The CR model has proven effective in addressing various social challenges in Japan, such as disaster prevention and relief, by leveraging local capacities and networks (26).

This article aims to advance knowledge on the integration of CR within the SE framework for dementia care in Japan. We discuss four key initiatives, which incorporate CR principles, we provide a deeper understanding of how community capacity-building and social capital can be harnessed to address social isolation and loneliness among PLwD. Furthermore, we discuss how Japan’s experience can offer valuable lessons for other countries in developing culturally tailored, community-based models to support PLwD.

## 2 Methodology

This study employs a case study methodology to explore community-based interventions aimed at reducing social isolation and loneliness in older adults with dementia in Japan. The analysis focuses on four key cases: (1) Community (Dementia) Cafés, (2) Dementia Supporter Caravan Support Training Program, (3) Community Social Worker (CSW), and the (4) Dementia Friendly-Community. Each case was selected based on its innovative approach to building social capital and community engagement within a socio-ecological framework.

Data for the case studies were gathered through a combination of document analysis and observations of programs. The data sources included government reports, program evaluations, and articles. The cases were analyzed to identify the mechanisms through which these interventions promote social inclusion and reduce the stigma surrounding dementia.

## 3 Four cases of SE approach

### 3.1 Service B community cafés as “third places” in dementia care

In Japan, the concept of Community Cafés was institutionalized through the public long-term care insurance program (PLTC). The PTLC was traditionally designed as a binary program composed of visiting home care or (outpatient) day care. However, revisions were made to the PTLC so that providers of visiting home care and day care services were widened to include volunteers and for-profits in addition to the traditional social service agencies. Under the “Service B,” a grant scheme increased local resident and volunteer initiated “day care services.” Service B grants allow food-based services, and the most represented model of service is community cafés. According to the long-term care preventive and daily living support comprehensive services guideline (27), community cafés are categorized as “outpatient services B (community-centered services)” which are expected to prevent long-term care needs and isolation, provide support for daily living, and increase older adults’ health and well-being.

The term Community Café was coined by a public corporation WAC (Wonderful Aging Club) led by a retired social welfare researcher. According to WAC, community cafés are “gathering place, a place of belonging in the communities… creating spaces where you feel at ease and connecting person and person are considered important.” Dementia cafés, a subset of Community Cafés, were promoted in June 2012 as a part of the 5-year national dementia policy planning. Dementia community cafés are diverse; there are community cafés which are open daily while others are open about once a month. Many places only charge a 100–200 Japanese yen (about $0.67 – $1.33 US dollar) for drinks and food. It is more than just a place for persons with dementia to congregate. There were7,904 are dementia cafés existed in 2021 (28).

Community Cafés, especially those targeting dementia care, are exemplary models of creating “third places”—spaces that are neither home (first place) nor workplace (second place) but are instead informal social environments that facilitate social interaction and foster a sense of community (28, 29). According to a national report on dementia cafés (30), expert opinions showed that the cafés served three purposes: a place for providing early detection and preventive care for dementia, a hub for making community connections, and a place for raising awareness and education on dementia. The “hub for making connections” included keywords such as “sense of belonging,” “prevent isolation,” and “a place to relax.” This aligns with the concept of “the third place,” which is neither one’s home nor workplace, but a space for informal, free social interaction that encourages a sense of warmth and conviviality (31). Interviews with spousal caregivers of PLwD who were participating in dementia cafés were asked about their caregiving experiences. Findings showed seven themes, which included loneliness as a key theme of their caregiving experience and the study acknowledged that dementia cafés were critical for alleviating their loneliness (32).

Writing to urban planners, Oldenburg suggests that “third places” work best when it is within walking distance and run locally and independently rather than commercially owned (33). Dementia community cafés in Japan are designed to be accessible and run by local volunteers and residents (28). Approximately over half of the cafés are held in long-term care or medical facilities (53%), followed by public facilities (18.4%), and cafés (12.8%), and if there are any access issues, some cafés offer car services (28). Unlike the more structured “salons,” which focus primarily on preventive care activities and services, dementia community cafés also emphasize secondary prevention by providing a space for early detection and support. Community cafés in Japan have played a crucial role for integrating PLwD into their communities, reducing stigma, providing preventive care, and promoting more inclusive social environments.

### 3.2 Ninchisho (dementia) supporter caravan

While the effectiveness of community-based interventions like Community Cafés is further enhanced by Japan’s concept of CR—the “power” or “capacity” of a community, the Dementia Supporter Caravan initiative is a notable example of how CR is operationalized to empower communities and enhance social capital. Launched in 2005 as part of the Ministry of Health, Labor, and Welfare’s “10-year Campaign to Understand Dementia and Build Community Networks,” the Dementia Supporter Caravan aims to raise public awareness and foster supportive networks for people with dementia (34). As part of this overarching effort, the ‘Dementia Supporter Caravan’ initiative was launched with the aim of educating community members, equipping them with knowledge about dementia, and establishing a network of supporters for PLwD and their families (35). This initiative has a three-step strategy (35). First, healthcare experts in dementia provide a 6-h training course to people involved in dementia consultation or care, transforming them into ‘caravan mates.’ Second, using a textbook and visual materials, these caravan mates conduct 90-min educational training sessions for the broader community, including residents, students, employees of various organizations, paid professional caregivers, and families and friends who care for PLwD. Those trained by the caravan mates are designated as dementia supporters. Finally, these dementia supporters organize themselves locally to strategize dementia training and education in their own communities. An orange bracelet is given to those who completed the training as a certificate at the end of the 90-min session.

By the end of March 2024, over 15 million individuals had become dementia supporters (36), demonstrating the initiative’s extensive reach. The program builds CR by mobilizing existing community resources, enhancing public understanding of dementia, and fostering empathy and solidarity among community members. The initiative’s success highlights the power of community-based education in shifting social norms, reducing stigma, and promoting inclusive and dementia-friendly communities.

The Dementia Supporter Caravan has also proven adaptable beyond Japan, with similar programs being implemented in other countries (37). This international uptake reflects the potential of CR as a scalable and adaptable model that can be integrated into different cultural contexts, provided there is a clear understanding of local conditions and community dynamics.

### 3.3 Community social work

Community Social Workers (CSWs) are integral to Japan’s efforts to address social isolation, particularly among older adults with dementia. In Osaka, proactive implementation of CSWs began in 2003, driven by the complexity of social and personal problems across various domains. A study of CSWs (*n* = 1,335) found that 74% worked with older adult households (38). Among cases of social isolation, 76.7% had limited contact with relatives or the community, with causes including mental illness (26.7%) and dementia (16.7%).

Scholarly debate has emphasized the uniqueness of Japanese community social work, rooted in Shigeo Okamura’s theories, which shaped Japan’s approach to addressing social issues through a symbiotic network of formal and informal support systems (39). Outreach is the core activity of CSWs. Katsube, a prominent CSW, describes their role as fostering mutual support and motivating residents through bottom-up solutions that increase CR (40).

The geographical focus of CSWs within clearly defined local areas is pivotal for effective outreach. Typically, a CSW covers a junior high school district, equating to a population size of 5,000 to 10,000 residents, or approximately a 30-min walking distance. This area demarcation is considered optimal for CSWs to deliver their services effectively, as it encompasses the daily living radius of many older adults (41). They collaborate with stakeholders such as municipal governments, community care centers, and volunteers. A key initiative is training local gatekeepers (mimamori supporters) to prevent social isolation and identify at-risk individuals, particularly PLwD (42). Gatekeepers are trained not only in early detection but also in reducing stigma and discrimination, emphasizing that dementia does not define the individual.

CSWs enhance community resilience by creating networks both within and beyond traditional circles. Their work reflects a bottom-up approach, co-creating solutions with residents, and aligning with CR to strengthen social capital, empathy, and self-governance within the community.

### 3.4 Omuta City dementia care community

A key factor in reducing social isolation and loneliness for PLwD is fostering a sense of belonging within the community. Social capital interventions must aim to build meaningful connections and emotional satisfaction to effectively combat loneliness. In Japan, this is achieved through dementia education programs and inclusive projects that redefine public spaces as welcoming environments for PLwD.

The Omuta City Dementia Care Community, known as the “Omuta Model,” is an example of how chiiki-ryoku (CR) can be leveraged to create inclusive communities for PLwD. Located in a former coal-mining town, Omuta City has a rapidly aging population, with 38% of its residents aged 65 or older in 2023 (43). The city launched a social experiment involving collaboration between the public and private sectors, multigenerational exchanges, and various local institutions such as businesses, media, transportation services, and schools (44, 45).

One key feature of the Omuta Model is the community-based SOS network to locate missing individuals with dementia. When a PLwD is reported missing, police share their information with local organizations, including taxi companies, post offices, and fire departments. Information is also sent to citizens via email. Annual simulations involve 2,000 participants, with 7% of the city’s population receiving training, compared to the national average of 3% (46). This bottom-up approach fosters trust, cooperation, and collective action within the community. 4 years of data on missing or wandering persons living with dementia (PLwD) who were protected by the network showed an overall increasing trend, with the highest increase of 39.7% occurring between 2011 (121 persons) and 2012 (169 persons) (46).

Another significant aspect is education. A dementia program for children, from 4th grade to junior high, promotes intergenerational solidarity by teaching students about dementia and how to support PLwD. Through role-play and group discussions, this dementia education program helps children gain knowledge about dementia and learn ways to support PLwD (47). About 6,000 children have participated in the program over the past decade (47). Additionally, healthcare professionals undergo 400 h of training to become dementia coordinators, with a curriculum covering dementia and human rights. Most dementia coordinators are nurses and social workers.

The Omuta Model has attracted national and international attention as an effective strategy to build a community where all individuals, including those with dementia, can live with dignity. It showcases the potential of integrating CR into public health strategies that focus on local governance and lifelong learning.

## 4 Discussion

Social capital interventions, which foster networks that enable individuals to access support, have been shown to impact health outcomes, including mental health and loneliness in older adults (48, 49). These interventions, which encourage social engagement and the development of robust networks, are recognized as potential preventive measures against cognitive decline (50). Research suggests that community engagement and social involvement help build cognitive reserve, potentially mitigating dementia-related brain changes (51, 52). However, the effectiveness of these interventions varies, depending on research designs, populations, and measurement tools (53, 54). Additionally, while increasing social interactions may alleviate loneliness, the depth and quality of relationships are crucial. Community-level interventions that align with local norms and involve members in program development and implementation are more likely to succeed. Such an approach can foster transformative change, both individually and collectively (55).

This study uses the socio-ecological (SE) model and chiiki-ryoku (CR) to examine community interventions designed to reduce social isolation among people living with dementia (PLwD) in Japan. The analysis reveals the importance of strategically defining intervention spaces, reimagining relationships and resources, and emphasizing local governance.

### 4.1 Defining spaces for social capital interventions

Strategically defining spaces is key to effective interventions. Japan’s Ministry of Health, Labor, and Welfare emphasizes spatially defined interventions in its “Vision for Provision of Social Services in the New Era” (56). Central to this is nichijo seikatsuken—the “everyday living area”—a geographic space within walking distance, representing the foundation of daily life. This approach provides a localized framework for interventions, ensuring they are pragmatic and context-sensitive.

For PLwD, as mobility declines, access to local resources and networks becomes vital (57). Studies show that well-defined local spaces, such as neighborhoods, tend to foster stronger social connections and support networks (58, 59). Smaller spatial units, like traditional associations and neighborhood groups, have proven effective (60–62), while larger units, like municipalities, tend to be less successful in building social capital (63). However, smaller spaces also pose challenges for PLwD, as stigma and discrimination can exacerbate isolation. Research shows that bonding social capital (close-knit ties) can place excessive obligations on residents, leading to social fatigue, whereas bridging capital (connections across groups) may have more positive health effects (64).

The four Japanese initiatives analyzed here focus on junior high school districts or municipal levels, allowing for the development of diverse relationships. This broader focus helps build bridging and linking social capital, creating a more supportive community (23, 65). Combining bonding, bridging, and linking social capital creates a holistic community environment, offering lessons for other countries to adapt culturally sensitive strategies for dementia care.

### 4.2 Reimagining the relationships and resources

Studies in the United States on senior centers as hubs for dementia-friendly initiatives emphasize the importance of social, human, and programmatic capital (66, 67). Similarly, our analysis of Japan’s initiatives highlights the role of social capital in reimagining community relationships through the training of community social workers and lay dementia supporters.

However, past studies often prioritize organizational characteristics over program quality and types of social engagement. Given our focus on social isolation and loneliness among PLwD and their families, we examined the types of social capital accessible to these groups.

In Japan, segmentation and channeling strategies support PLwD and their families by adapting to local cultural contexts. Segmentation targets specific sub-groups, ensuring interventions reach those who benefit most, while channeling uses social capital to link interventions with desired outcomes (68). Service B Dementia Cafés, for example, adapt to local contexts, and community social workers connect marginalized individuals to broader community networks, breaking down silos in care. This mobilizes social capital to create safety nets for PLwD and their caregivers.

Both the Dementia Supporter Caravan and the Omuta Care Community Model channel formal and informal education to cultivate empathic, supportive networks for individuals with dementia. The strategic use of social capital through lifelong learning has been successful. Japan’s New Orange Plan, with goals set for 2025, has surpassed five of its 11 targets as of 2017 (69). Over 15 million people have participated in the Dementia Supporters Caravan, nearly double the number from 2014. These initiatives reinforce knowledge and social capital, promoting inclusivity for individuals with dementia.

The Omuta Model’s approach to community capacity-building engages socially isolated individuals, showing that enhancing social capital can foster a more inclusive community. Research by Murayama et al. highlights the positive correlation between strong community social capital and cognitive health, suggesting that communities with robust ties have lower rates of cognitive decline, reinforcing the importance of a dementia-friendly society (70).

The four Japanese initiatives take a multi-system approach, targeting social, human, and tangible capital to reach a wider demographic. Japan’s comprehensive policies offer valuable insights for countries like the U.S., where strong social safety nets can be paired with individualism. Enhanced coordination and resource allocation for dementia care could reduce long-term costs associated with advanced stages of the disease.

### 4.3 Emphasis on local governance

Previous literature on community-level preventive efforts for cognitive health often advocates for socio-ecological (SE) approaches that create a sense of belonging and enable older adults to “age in place” with a sense of “at-homeness” (71). However, our analysis challenges this assumption, revealing that many Japanese older adults experience stigmatization within their communities, lacking such sentiments. A more dynamic strategy is needed, where social relations are actively managed rather than passively maintained.

In addition to social and human capital, chiiki-ryoku (CR) emphasizes the critical role of local governance. This approach empowers individuals and communities to become proactive agents of change. The Japanese government recognizes that service delivery depends on raising awareness and empowering community members. In a culture where social welfare is often seen as someone else’s problem, CR promotes a shift in perspective, encouraging individuals to take ownership of community matters (marugoto) as personal responsibility (wagagoto). This shift in community efficacy, or belief in the ability to enact change, is crucial for addressing complex issues like social isolation and loneliness. Strengthening local governance enhances a community’s capacity to respond, making it more resilient, particularly in crises like the COVID-19 pandemic. Japan’s model shows how local governance and community empowerment mitigate social isolation and promote inclusive support, especially for older adults with dementia.

The four initiatives highlight how CR, through local governance, multi-system approaches to social capital, and community capacity-building, fosters resilience to prevent and mitigate isolation among PLwD. This approach addresses a critical dimension missing in many interventions: the chronic nature of social isolation and its long-term health consequences (72). Golubchikov’s concept of “persistent resilience” emphasizes the need for flexible, ongoing adaptation to society’s unpredictable challenges (73). He describes ‘persistent resilience’ as having four dimensions: relational, dynamic, spatial, and political. Strengthening these dimensions can catalyze transformation at both individual and collective levels. Japan’s initiatives show promise in addressing these four dimensions to prevent social isolation among PLwD.

The four initiatives demonstrate that CR, with its emphasis on local governance, a multi-system approach to social capital, and community capacity building, effectively fosters community resilience to prevent and mitigate social isolation and loneliness among persons living with dementia (PLwD). This approach highlights a critical dimension often missing in current interventions: the chronic nature of social isolation and loneliness among PLwD, and their serious long-term health consequences for older adults. Golubchikov’s temporal perspective, emphasizing the unpredictability of contemporary society, underscores the inadequacy of traditional community-level interventions (73). There is an increasing need for ongoing resilience and flexible adaptation to navigate persistent challenges. Golubchikov describes this as ‘persistent resilience,’ encompassing four key dimensions: relational, dynamic, spatial, and political (73). Strengthening these dimensions can catalyze transformation at both individual and collective levels. Japan’s initiatives notably address these four dimensions, showing promise in preventing and mitigating social isolation and loneliness among PLwD.

This article examines how community-based interventions in Japan can help alleviate social isolation and loneliness among PLwD. It highlights the socio-ecological (SE) approach, which leverages social capital through community-level initiatives like Community Cafés, Dementia Supporter Caravan, Community Social Workers, and the Omuta City Dementia Care Model. These interventions incorporate the culturally grounded concept of CR to foster community resilience, reduce stigma, and create inclusive environments for PLwD and their caregivers. These initiatives provide a foundation for mitigating loneliness through community engagement and emphasize community-driven strategies as essential in addressing the social needs of PLwD.

The article highlights several limitations that must be addressed. Empirical evidence supporting the effectiveness of community-based (CR) interventions is currently limited, and future research should rigorously assess the long-term impact of these programs on reducing social isolation and improving dementia care outcomes. Additionally, there is a need for a deeper understanding of how the socio-ecological approach can be scaled up and adapted across different cultural contexts while maintaining its effectiveness. CR interventions may be more naturally suited to countries that emphasize communality, but they also offer personal benefits in more individualistic contexts, such as fostering personal fulfillment, social connection, cognitive health, and other individual goals.

CR-based activities like education and community network building appeal to a wide range of individuals, including those who are socially active and thriving, as the risk of social isolation and loneliness can affect anyone. The reciprocity within these programs, and their focus on bridging rather than bonding social capital beyond close-knit communities, may also resonate with more individualistic societies. The flexible, autonomous, and reciprocal nature of these interventions could provide personal advantages even in less collective environments.

Furthermore, the article underscores the importance of continued investment in research to refine context-specific community models and multi-level strategies, enhancing our understanding of how social capital interventions can be optimized for diverse populations. Addressing these limitations will be crucial for developing evidence-based practices that support persons living with dementia (PLwD) globally, ensuring that interventions are not only culturally sensitive but also sustainable and scalable.

## Data Availability

The original contributions presented in the study are included in the article/supplementary material, further inquiries can be directed to the corresponding author.
